# Phantom Breast Syndrome

**DOI:** 10.4103/0973-1075.58453

**Published:** 2009

**Authors:** Ramesh, Nootan K Shukla, Sushma Bhatnagar

**Affiliations:** Department of Surgical Oncology, Dr. BRA Institute Rotary Cancer Hospital, All India Institute of Medical Sciences, New Delhi, India; 1 Department of Anesthesiology, Dr. BRA Institute Rotary Cancer Hospital, All India Institute of Medical Sciences, New Delhi, India

**Keywords:** Breast cancer, Phantom breast syndrome, Quality of life

## Abstract

Phantom breast syndrome is a type of condition in which patients have a sensation of residual breast tissue and can include both non-painful sensations as well as phantom breast pain. The incidence varies in different studies, ranging from approximately 30% to as high as 80% of patients after mastectomy. It seriously affects quality of life through the combined impact of physical disability and emotional distress. The breast cancer incidence rate in India as well as Western countries has risen in recent years while survival rates have improved; this has effectively increased the number of women for whom post-treatment quality of life is important. In this context, chronic pain following treatment for breast cancer surgery is a significantly under-recognized and under-treated problem. Various types of chronic neuropathic pain may arise following breast cancer surgery due to surgical trauma. The cause of these syndromes is damage to various nerves during surgery. There are a number of assumed factors causing or perpetuating persistent neuropathic pain after breast cancer surgery. Most well-established risk factors for developing phantom breast pain and other related neuropathic pain syndromes are severe acute postoperative pain and greater postoperative use of analgesics. Based upon current evidence, the goals of prophylactic strategies could first target optimal peri-operative pain control and minimizing damage to nerves during surgery. There is some evidence that chronic pain and sensory abnormalities do decrease over time. The main group of oral medications studied includes anti-depressants, anticonvulsants, opioids, N-methyl-D-asparate receptor antagonists, mexilitine, topical lidocaine, cannabinoids, topical capsaicin and glysine antagonists. Neuromodulation techniques such as motor cortex stimulation, spinal cord stimulation, and intrathecal drug therapies have been used to treat various neuropathic pain syndromes.

## INTRODUCTION

One in eight women will develop breast cancer of which approximately 60% are treated surgically for axillary node staging and primary breast tumor resection. It is estimated that over 50% of women suffer chronic pain following treatment for breast cancer surgery. “Phantom Breast Syndrome” (PBS) is a type of condition in which patients have a sensation of residual breast tissue and can include both non-painful sensations as well as phantom breast pain. Patient may have pain and discomfort, itching, pins and needles sensations, tingling, pressure, burning, and throbbing. The syndrome can start even after more than one year of surgery. The incidence varies across different studies, ranging from approximately 30% to as high as 80% of patients after mastectomy.[1] PBS can persist years after surgery.[2] It seriously affects quality of life through the combined impact of physical disability and emotional distress. A recent report presented at the American Society of Anesthesiologists meeting demonstrated that depression and fear of cancer recurrence was greater in women who reported phantom pain. Those women also had more concerns than others that the mastectomy would have an impact on their sex lives. The breast cancer incidence rate in India as well as Western countries has risen in recent years while survival rates have improved; this has effectively increased the numbers of women for whom post-treatment quality of life is important. In this context, chronic pain following treatment for breast cancer surgery is a significantly under-recognized and under-treated problem. Neuropathic pain is the most prevalent type of pain and it may be derived from the breast cancer, breast cancer surgery and non-surgical treatment. The surgery-related pain syndromes present as pain in the surgical scar, chest wall and upper arm, as well as shoulder discomfort and phantom breast dysesthesias and paresthesias. Other neuropathic pain syndromes that may add to functional impairment include tumor recurrence pain, paraneoplastic processes, complex regional pain syndrome, chemotherapy-associated neuropathy (especially paclitaxel), radiation plexitis and plexopathy.

## DISCUSSION AND REVIEW OF THE LITERATURE

In a recent review, Jung *et al.*,[3] found that the literature was inconsistent in defining chronic pain after breast cancer surgery. Earlier studies analyzing prevalence did not distinguish between the various neuropathic pain syndromes. To improve comparability between future studies, Jung *et al.*,[3] suggested a consistent timeframe definition of chronic neuropathic pain and syndrome classification based on potential etiology. They further suggested that neuropathic pain syndromes due to breast cancer surgery should be considered chronic after three months and that shorter timeframes should raise a consideration of pain associated with tumor recurrence. Jung *et al.*,[3] distinguished four different types of chronic neuropathic pain due to surgical trauma following breast cancer surgery: (1) Phantom Breast Pain is pain experienced in the area of a removed breast, (2) Intercostobrachial Neuralgia is pain often accompanied with sensory changes, in the distribution of the intercostobrachial nerve following breast cancer surgery with or without axillary dissection. The intercostobrachial nerves run from the chest wall through the axilla to innervate the shoulder and upper arm. With axillary node dissection, these nerves are impossible to spare. Unfortunately, the risk of damage to the intercostobrachial nerve in breast-conserving surgery can be at times equivalent to that which occurs with complete mastectomy. Granek *et al.*,[4] revealed a wide variation in the size, location and branching of the intercostobrachial nerve which may explain the high risk of damage to these nerves irrespective of the surgical approach. Sensory symptoms have been shown to vary depending on the origin at which the nerve is sectioned. Post-mastectomy pain syndrome (PMPS) consists of pain and sensory changes localized to the axilla, medial upper arm, and/or the anterior chest wall on the ipsilateral side of the surgery. Pain starts immediately or soon after breast surgery. Damage to the intercostobracial nerve has been identified as the most common cause of PMPS.[1,6] Incidence varies from 20-50%.[3] Neuroma pain (including scar pain) is pain in the region of a scar on the breast, chest, or arm that is provoked or exacerbated by percussion. A neuroma is formed from masses of tangled axons formed at the end of severed peripheral nerves. Neuromas trapped in scar tissue have been shown to cause chronic neuropathic pain, spontaneous pain and severe sensitivity to pressure on the breast surgery area. Excision to enable relocation of the neuroma to a protected site may be beneficial, but may risk an increase in neuropathic pain.[4] Other nerve injury pain may result from damage or traction to the medial and lateral pectoral, long thoracic, or thoracodorsal nerves. In Jung *et al.*'s review, there were 21 studies with follow-up periods from 1-96 months (one study of 210 months), which revealed the following widely varying ranges of prevalence estimates: Phantom breast pain 3-44%, intercostobrachial neuralgia (ICN) 16-39% for all breast cancer surgery, ICN in breast-conserving surgery 14-61% and neuroma pain 23-49%.[3] Trial sizes to date have ranged from 22 to 283 patients, leaving considerable uncertainty about the overall size of the problem. However, even estimates at the lower end of these ranges suggest that the problem is considerable. Variations in the reported size of the problem are also due to other factors including duration of time since surgery, type of surgery, research method, diagnostic criteria, pain assessment methods, and the distribution of various demographic and clinical characteristics in the samples studied. In addition, very few studies differentiate pain syndromes according to the type of surgical procedure used. Wallace *et al.*,[6] analyzed the incidence, intensity and character of pain after four types of breast surgery: Mastectomy for breast cancer; mastectomy with reconstruction for breast cancer; cosmetic augmentation; and breast reduction. The highest incidence occurred in the combined mastectomy and reconstruction with breast implants at 53%. There was equal incidence of about 30% in those undergoing reconstruction without implants and mastectomy without reconstruction. Breast augmentation with subglandular implants and breast reduction were the lowest at 21%. In a recent study, Dijkstra *et al.*,[7] found the prevalence of phantom breast sensations or pain to be lower than in most previous studies. They attributed these differences to research methodology. In comparing their methods with those of the accompanying literature review of 29 studies, they found that prospective studies on an average showed higher prevalence of phantom breast pain and lower prevalence of phantom breast sensation compared to cross-sectional studies. Data collection by interview revealed lower prevalence in both compared to questionnaires.

There are a number of assumed factors causing or perpetuating persistent neuropathic pain after breast cancer surgery. There is, however, a lack of large-scale multiple risk factor studies identifying the variables as independent risk factors or evaluating their relationships with other variables, which are known to affect the development of chronic pain. From the literature currently available, the most well-established risk factors for developing phantom breast pain and other related neuropathic pain syndromes are severe acute postoperative pain and greater postoperative use of analgesics.[8,9] These are consistent with all persistent post-surgical neuropathic pain syndromes. Hence, it is assumed that the relief of severe acute pain may reduce the risk of chronic pain. Preoperative breast pain correlated with increased phantom breast sensation and phantom breast pain.[10]

Underlying each of the four classifications of pain after breast cancer surgery is damage to various nerves during surgery. Nerve preservation approaches have shown reduced incidence of sensory deficits (53% vs 84% of women) but nerve-sparing is only successful in 65% of the cases where it was attempted.[11] Evidence to support age as a risk factor is currently inconclusive. Younger patients (under 35 years of age), however, have poorer prognosis due to more aggressive cancers or higher rates of recurrence. Chemotherapy and radiation therapy are reported not to be direct risk factors of phantom breast pain but may cause additional pain through peripheral neuropathy, plexopathy, and plexitis. Psychosocial distress has been found to be both a consequence of chronic pain and a risk factor for its development.[8] While younger age and being unmarried were also independently associated with persisting acute pain, these were postulated to reflect the psychosocial effects of reduced social support.

Phantom breast pain and other pain syndromes severely affect the quality of life of patients. The negative impact on a patient's physical and psychosocial functioning is consistent with many chronic and cancer pain syndromes. It has been reported that up to half of patients report negative impact of pain on their activities and up to one-quarter report moderate to high impact on their daily activities at home and work. Not surprisingly, studies have also found that breast cancer surgery patients with chronic pain have a greater psychological stress and psychiatric morbidity than the general population.[8,12]

**Figure 1:**
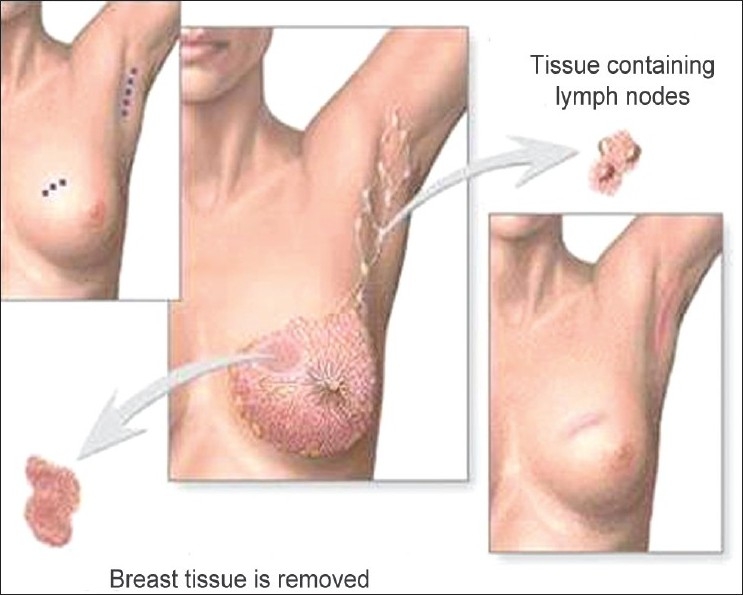
Breast conservation surgery

**Figure 2:**
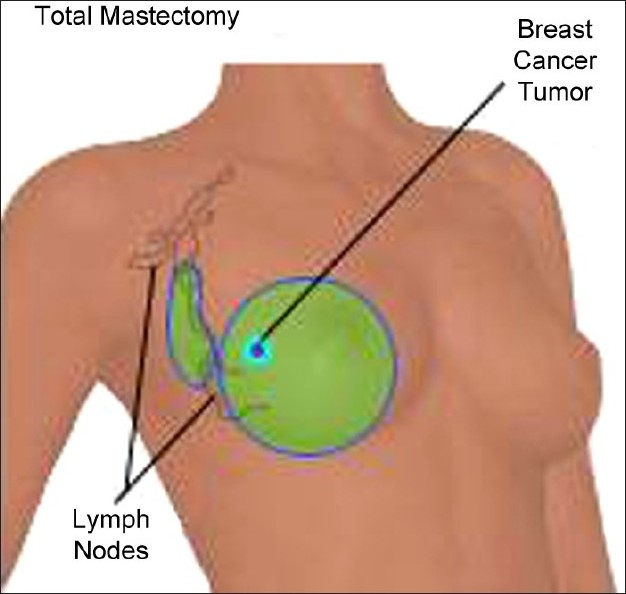
Modified radical mastectomy

**Figure 3:**
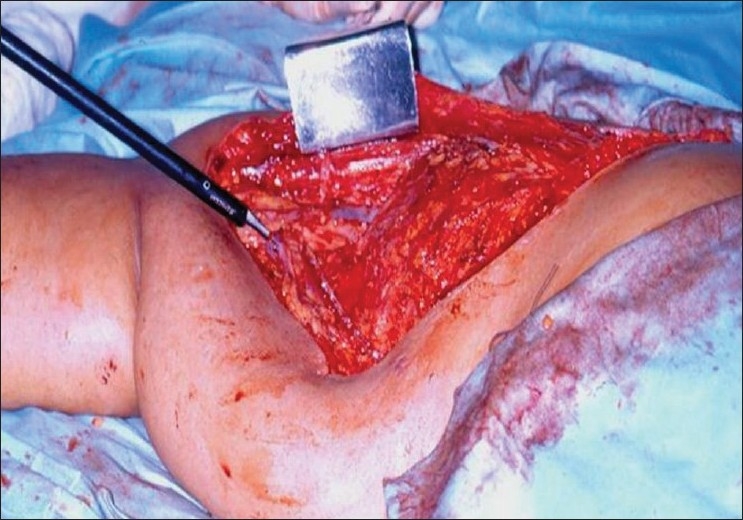
Modified radical mastectomy: Intraoperative photograph

Based upon current incomplete evidence, the goals of prophylactic strategies could first target optimal perioperative pain control and minimizing damage to nerves during surgery. Peri-operative Pain Control: Medications traditionally used for persistent neuropathic pain such as topical EMLA,[13] gabapentin[14] and mexilitine[14] have been used in patients undergoing breast cancer surgery and have been reported in some studies to have benefits in reducing acute postoperative pain, one of the identified risk factors. However, more effective treatment regimens need to be evaluated. A broad-based approach targeting the mechanisms involved in persistent neuropathic pain after breast cancer surgery is also required. Minimizing damage to nerves during surgery: Improved screening methods detect breast cancer at earlier stages. Earlier detection means smaller tumor sizes, which has made breast-conserving surgical treatments [Figure 1] possible and widely used over the traditional method, modified radical mastectomy [Figures 2 and 3]. These currently account for up to 40% of breast cancer surgery.[15] Breast-conserving techniques include lumpectomy, conservative breast surgery, wide local excision, partial mastectomy, segmentectomy, or tylectomy. Such approaches include reducing the number of axillary dissections required. Combining reduced surgical trauma with nerve preservation techniques may reduce the risk of sensory deficits and the occurrence of ICN.[16,17] In this regard, the increased use of less invasive staging techniques such as sentinel lymph node biopsy has helped to reduce the number of patients undergoing axillary dissection and the resulting trauma to intercostobrachial nerves.[18]

There is some evidence that chronic pain and sensory abnormalities do decrease over time.[19] Unfortunately, there are very few studies looking at the natural history of pain duration of individual neuropathic pain syndromes. Most are retrospective studies using data combined from all sources of pain following surgery. In the best existing population-based study of long-term outcomes, Macdonald *et al.*,[20] found that 7-12 years post-surgery, 52% of women who had PMPS at four-year follow-up still had PMPS; these women had significantly lower quality of life compared with those women whose PMPS had resolved.

Well-established PBS needs treatment. In 2005, Finnerup *et al.*,[21] reviewed all randomized, doubled-blinded, placebo controlled trials for evidence to support a neuropathic pain treatment algorithm. One hundred and five trials were included covering a number of neuropathic conditions. The main groups of oral medications studied included anti depressants, anticonvulsants, opioids, NMDA antagonists, mexilitine, topical lidocaine, cannabinoids, topical capsaicin, and glycine antagonist.

For persistent post-breast cancer surgery neuropathic pain, there exist very few randomized, double-blind, placebo-controlled trials. The use of topical capsaicin[22,23] or amitryptiline[24] has reported benefit in the treatment of pain in patients after breast cancer surgery.

However, many unanswered questions remain about the optimal doses, timing and coordination of therapy with ongoing adjuvant treatment for breast cancer. There are also a number of medications and multidisciplinary approaches showing benefit for other types of neuropathic pain that have yet to be trialed. In women with early-onset or established post-breast cancer surgery neuropathic pain, neuromodulation techniques such as motor cortex stimulation,[25,26] spinal cord stimulation[27] and intrathecal drug therapies[28] have been used to treat various neuropathic pain syndromes. Early case reports of the use of peripheral nerve stimulation[29] for persistent neuropathic pain syndromes such as occipital neuralgia, trigeminal postherpetic neuralgia, and trigeminal post-traumatic neuropathic pain may hold promise for its use in post-surgical pain syndromes. To date, there have been no reported studies in this area.

## FUTURE DIRECTIONS

The small scale of existing studies in persistent pain after breast cancer surgery creates considerable uncertainty regarding the generalizability of their findings, and also regarding the identification of potentially modifiable risk factors. Recent improvements in neuropathic pain screening tools now make early identification and syndrome classification of neuropathic pain more achievable.[30] Many important questions remain about persistent neuropathic pain after breast cancer surgery including the natural history, the predisposing risk factors, current awareness and management approaches by treating surgeons and oncologists. Various studies are going on to explore the size of the problem, their associated factors, detection and optimal management of persistent pain after breast cancer surgery. These study outcomes will be used to establish guidelines for providing information to patients, timely diagnosis of pain by treating surgeons and oncologists and early pain management. On a broader level, these studies will also help us identify the issues affecting optimal access and use of pain management centers in general.

## References

[1] Staps T, Hoogenhout J, Wobbes T (1985). Phantom breast sensations following mastectomy. Cancer.

[2] Kroner K, Knudsen UB, Lundby L, Hvid H (1992). Long – term phantom breast syndrome after mastectomy. Clin J Pain.

[3] Jung BF, Ahrendt GM, Oaklander AL, Dworkin RH (2003). Neuropathic pain following breast cancer surgery: Proposed classification and research update. Pain.

[4] Granek I, Ashikari R, Foley K (1984). The post-mastectomy pain syndrome: Clinical and anatomical correlates. Proc Am Soc Clin Oncol.

[5] Vecht CJ, Van de Brand HJ, Wajer OJ (1989). Post-axillary dissection pain in breast cancer due to a lesion of the intercostobrachial nerve. Pain.

[6] Wallace MS, Wallace AM, Lee J, Dobke MK (1996). Pain after breast surgery: A survey of 282 women. Pain.

[7] Dijkstra PU, Rietman JS, Geertzen JH (2007). Phantom breast sensations and phantom breast pain: A 2-year prospective study and a methodological analysis of literature. Eur J Pain.

[8] Tasmuth T, Estlanderb AM, Kalso E (1996). Effect of present pain and mood on the memory of past postoperative pain in women treated surgically for breast cancer. Pain.

[9] Tasmuth T, Kataja M, Blomqvist C, von Smitten K, Kalso E (1997). Treatment-related factors predisposing to chronic pain in patients with breast cancer: A multivariate approach. Acta Oncol.

[10] Kroner K, Krebs B, Skov J, Jorgensen HS (1989). Immediate and long-term phantom breast syndrome after mastectomy: Incidence, clinical characteristics and relationship to pre-mastectomy breast pain. Pain.

[11] Abdullah TI, Iddon J, Barr L, Baildam AD, Bundred NJ (1998). Prospective randomized controlled trial of preservation of the intercostobrachial nerve during axillary node clearance for breast cancer. Br J Surg.

[12] Akechi T, Okuyama T, Imoto S, Yamawaki S, Uchitomi Y (2001). Biomedical and psychosocial determinants of psychiatric morbidity among postoperative ambulatory breast cancer patients. Breast Cancer Res Treatment.

[13] Fassoulaki A, Sarantopoulos C, Melemeni A, Hogan Q (2000). EMLA reduces acute and chronic pain after breast surgery for cancer. Regional Anaesthesia Pain Med.

[14] Fassoulaki A, Patris K, Sarantopoulos C, Hogan Q (2002). The analagesic effect of gabapentin and mexiletine after breast surgery for cancer. Anesth Analg.

[15] Iglehart JD, Kaelin CM, Townsend CM (2001). Diseases of the breast in Sabiston textbook of surgery.

[16] Rietman JS, Geertzen JH, Hoekstra HJ, Baas P, Dolsma WV, de Vries J (2006). Long term treatment related upper limb morbidity and quality of life after sentinel lymph node biopsy for stage I or II breast cancer. Eur J Surg Oncol.

[17] Peintinger F, Reitsamer R, Stranzl H, Ralph G (2003). Comparison of quality of life and arm complaints after axillary lymph node dissection vs sentinel lymph node biopsy in breast cancer patients. Br J Cancer.

[18] Lyman GH, Giuliano AE, Somerfield MR, Benson AB, Bodurka DC, Burstein HJ (2005). American Society of Clinical Oncology Guideline Recommendations for Sentinel Lymph node biopsy in early-stage breast cancer. J Clin Oncol.

[19] Ivens D, Hoe AL, Podd TJ, Hamilton CR, Taylor I, Royle GT (1992). Assessment of morbidity from complete axillary dissection. Br J Cancer.

[20] Macdonald L, Bruce J, Scott NW, Smith WC, Chambers WA (2005). Long-term follow-up of breast cancer survivors with post mastectomy pain syndrome. Br J Cancer.

[21] Finnerup NB, Otto M, McQuay HJ, Jensen TS, Sindrup SH (2005). Algorithm for neuropathic pain treatment: An evidence based proposal. Pain.

[22] Watson CP, Evans RJ, Watt VR (1992). The post-mastectomy pain syndrome and topical capsaicin: A randomized trial. Pain.

[23] Dini D, Bertelli G, Gozza A, Forno GG (1993). Treatment of the post-mastectomy pain syndrome with topical capsaicin. Pain.

[24] Kalso E, Tasmuth T, Neuvonen PJ (1995). Amitriptyline effectively relieves neuropathic pain following treatment of breast cancer. Pain.

[25] Brown J, Tilitsis J (2006). Motor cortex stimulation. Pain Med.

[26] Nuti C, Peyron R, Garcia-Larrea L, Brunon J, Laurent B, Sindou M (2005). Motor cortex stimulation for refractory neuropathic pain: Four year outcome and predictors of efficacy. Pain.

[27] De Andres J, van Buyten JP (2006). Neural Modulation by Stimulation. Pain Practice.

[28] Hassenbusch SJ, Portenoy RK, Cousins M, Buchser E, Deer TR, Du Pen SL (2004). Polyanalgesic Consensus Conference 2003: An update on the management of pain by intraspinal drug delivery-report of an expert panel. J Pain Symptom Manage.

[29] Johnson M, Burchiel K (2004). Peripheral stimulation for treatment of trigeminal postherpetic neuralgia and trigeminal posttraumatic neuropathic pain: A pilot study. Neurosurgery.

[30] Bennet M, Johnson M, Oxberry S, Robb K, Simpson KH (2007). Using screening tools to identifiy neuropathic pain. Pain.

